# Childhood socioeconomic status and adulthood dietary diversity among Indonesian adults

**DOI:** 10.3389/fnut.2022.948208

**Published:** 2022-09-23

**Authors:** Emyr Reisha Isaura, Yang-Ching Chen, Shwu-Huey Yang

**Affiliations:** ^1^Department of Nutrition, Faculty of Public Health, Airlangga University, Surabaya, Indonesia; ^2^School of Nutrition and Health Sciences, College of Nutrition, Taipei Medical University, Taipei, Taiwan; ^3^Research Group of Food Safety and Food Security, Faculty of Public Health, Airlangga University, Surabaya, Indonesia; ^4^Department of Family Medicine, School of Medicine, College of Medicine, Taipei Medical University, Taipei, Taiwan; ^5^Department of Family Medicine, Taipei Medical University Hospital, Taipei, Taiwan; ^6^Nutrition Research Center, Taipei Medical University Hospital, Taipei, Taiwan; ^7^Research Center of Geriatric Nutrition, College of Nutrition, Taipei Medical University, Taipei, Taiwan

**Keywords:** dietary diversity, adult, childhood, socioeconomic status, Indonesian

## Abstract

Food insecurity problems still exist among people in low-to-middle income countries. The long-term disadvantages of socioeconomic status may contribute to chronic food insecurity. However, whether childhood socioeconomic status factors are related to food insecurity in adulthood remains unclear. Thus, the aim of this study was to test the association between childhood socioeconomic status factors and one of the proxies for adulthood food security, dietary diversity. This study used the 2014 RAND Indonesia Family Life Survey dataset with 22,559 adult participants as study samples. The childhood socioeconomic status factors consisted of 16 questions about the participants’ conditions when they were 12 years old. Adult dietary diversity was assessed using the United Nations World Food Programme’s food consumption score. A linear regression model was used to analyze the association between variables. This study found that the number of owned books (β coef.: 3.713–7.846, *p* < 0.001), the use of safe drinking-water sources (β coef.: 0.707–5.447, *p* < 0.001–0.009) and standard toilets (β coef.: 1.263–4.955, *p* < 0.001–0.002), parents with the habit of alcohol consumption (β coef.: 2.983, *p* = 0.044) or the combination with smoking habits (β coef.: 1.878, *p* < 0.001), self-employed with the permanent worker (β coef.: 2.904, *p* = 0.001), still married biological parents (β coef.: 1.379, *p* < 0.001), the number of rooms (β coef.: 0.968, *p* < 0.001), people (β coef.: 0.231, *p* < 0.001), and younger siblings (β coef.: 0.209–0.368, *p* < 0.001–0.039) in the same house were positively and significantly associated with the outcome variable. Furthermore, in the order of childhood socioeconomic status factors, self-employment without permanent workers and casual work types (β coef.: –9.661 to –2.094, *p* < 0.001–0.001), houses with electricity facilities (β coef.: –4.007, *p* < 0.001), and parents with smoking habits (β coef.: –0.578, *p* = 0.006) were negatively and significantly associated with the food security proxy. In conclusion, childhood and early socioeconomic disadvantage is related to adult food security status and may lead to poor health.

## Introduction

The Indonesian population who lived below the national poverty line in 2021 was around 10% (1, 2). The proportion of the employed population earning less than one dollar ninety cents purchasing power parity per day decreased from 10.4% in 2013 to 3.5% in 2019 (1). Besides the income level used to assess poverty, a previous researcher suggested child malnutrition as one of the poverty indicators (3). Meanwhile, Indonesia faces a child malnutrition problem called the “double burden of malnutrition” (4). For example, 24 children die for every 1,000 babies born in Indonesia in 2020 before they reach their fifth birthday (2).

Furthermore, socioeconomic status is related to people’s health status. However, economic situations such as poverty are related to a public health problem called food insecurity (5, 6). Thus, Indonesia is still dealing with food insecurity problems (6–8). Food insecurity is defined as an individual’s hardship in maintaining a healthy and active life with the consumption of a nutritious and balanced diet (9, 10). Another definition of food insecurity is a person’s complex situation that maintains the sustainability of food availability, food accessibility, and utilization of food for them to live an active and healthy lifestyle (11). Therefore, people who live below the poverty line or have food insecurity are more likely to have difficulty providing a nutritious and balanced diet for themselves and their families.

Food insecurity has three types based on its duration: chronic, transitory, and seasonal (12, 13). Chronic food insecurity is mainly due to persistent and long-term causes such as poverty. The impact of food insecurity on a person’s life includes the potential for adverse physical or mental health outcomes (7, 14–16). Furthermore, the long-term disadvantage in socioeconomic status may also contribute to chronic food insecurity (17, 18). Parents’ long-term income volatility and economic deprivation contribute to their children’s socioeconomic and food security status. Moreover, socioeconomic status during childhood is associated with adult health status and behavior (19–22). However, it is still unclear which socioeconomic status factors during childhood are related to adulthood food insecurity.

Conversely, food insecure people who live in urban areas and low-income people are considered vulnerable targets of the food insecurity intervention program. Food security assessment includes the following three pillars: availability, accessibility, and utilization of food (23). Food-secure people are more likely to consume more diverse foods because they have access to many available food types and their bodies can utilize foods well (24, 25). Dietary diversity is positively associated with food security pillars (24, 26). Dietary diversity is a qualitative measurement of food consumption that reflects the variety of foods accessed and is a proxy for nutrient adequacy (27). Meanwhile, one of the food security measurements, the food consumption score, considers dietary diversity and food frequency in composite score analysis (28, 29). Thus, we aimed to test the association between childhood socioeconomic status factors and food consumption scores.

## Materials and methods

### Dataset

This study used data from the Indonesia Family Life Survey (IFLS) by the RAND Corporation. The first IFLS collected samples representing approximately 80% of Indonesia’s population in 1993 (30). We used the fifth wave of the RAND-IFLS 2014 datasets. We included the basic information of the participants, such as anthropometric measurements, dietary information based on the 7 days before the survey, and information related to their health status. Our sample participants were those with complete data, were not breastfed or pregnant, did not have any disabilities, and were never diagnosed with cancer to minimize the analysis bias. This study included 22,559 adult participants aged 18–64 years. Trained nurses collected anthropometric (e.g., height, body weight, and waist circumference for the participants ≥ 40 years) and health-related data (i.e., blood pressure). The body mass index calculation uses body weight and height and is categorized into three groups based on the Indonesian BMI cutoff points (31). Abdominal obesity was defined as having a waist circumference cutoff point larger than 90 and 80 cm for men and women, respectively. Furthermore, we calculated physical activity assessment using the frequency, duration, and metabolic equivalent of task (MET) scores for each type of physical activity intensity (32). The data selection process is illustrated in Supplementary Figure 1.

### Childhood socioeconomic status factors and the outcome variable

The 2014 Indonesian Family Life Survey questionnaire included questions on childhood socioeconomic status. The assessment of childhood socioeconomic status uses subset questions from the China Health and Retirement Longitudinal Study (CHARLS) (30, 33). The childhood socioeconomic status questionnaire consists of information about the parents’ behavior, family, and housing situation when participants were 12 years old. The family situation section consisted of three questions about who lived with the participant, whether they lived with their biological mother or father, or whether their parents were still married. The housing situation questions include electricity and a standard toilet facility, drinking water source type, number of rooms, and people and siblings who lived in the same dwelling (30). A list of questions is presented in Supplementary Table 1. Childhood socioeconomic variables use categorical data except for the variables of the number of rooms, people, and siblings.

The aim of this study was to examine the association between socioeconomic status during childhood and food security proxy dietary diversity during adulthood. Furthermore, our study outcome variable is the food consumption score because the use of the food frequency questionnaire is relevant for food security assessment if defined by dietary diversity and food frequency (24, 29). The food consumption score used continuous data for statistical analysis. The United Nations World Food Programme (WFP) has developed a way to analyze food security using the dietary diversity proxy approach, called food score analysis, which results in a food consumption score (29). This study’s analysis of food scores used the number of days of ten eaten food types listed in the IFLS Food Frequency Questionnaire (FFQ). The steps to analyze food scores were to group the 10 food types into five groups (i.e., staples, protein, dairy products, fruits, and vegetables) and multiply the days of the food types with the score weighted for each food group. The weighted score is based on the food group’s nutrient density. The next step is to summarize the food group scores into scores for food consumption, which are further categorized into three levels of food consumption (i.e., poor, borderline, and acceptable) and two classes of food security (15, 29). The food security classes are food secure if the score falls within the acceptable level and food insecure if the score falls within the poor and borderline score.

### The ethical matter and statistical analysis

The study was conducted in accordance with the guidelines of the Declaration of Helsinki and approved by the Institutional Review Board of the RAND Corporation in the United States and the University of Gajah Mada in Indonesia. The RAND’s Human Subjects Protection Committee (RAND IRB) gave IFLS5 s0064-06-01-CR01. Informed consent was obtained from all participants involved in the study prior to data collection from the Indonesian Family Life Survey. Secondary data from the RAND-IFLS 2014 were used, and adult participants’ characteristics were reported as numbers (percentages) for categorical data and mean ± standard deviation for continuous data. Chi-square tests and *t*-tests were used to report the participants’ characteristics. We used a linear regression model to assess the association between food consumption and childhood socioeconomic status variables. The results of the linear regression are presented as coefficients and confidence intervals. Adjustments for the regression model included age and sex variables. Furthermore, *a p*-value < 0.05 was set as statistically significant. We used the STATA statistical software (v17.1; Stata Corp. LP, College Station, TX, USA).

## Results

### Study participants’ characteristics

The aim of this study was to assess the association between childhood socioeconomic status and adulthood food security proxy using RAND-IFLS 2014 secondary data. The total number of participants was 22,559, including 11,594 men and 10,965 women. The mean age of the participants was 38 ± 12 years, while the mean age of the men and women was 37 ± 12 and 38 ± 13 years, respectively. As shown in Table 1, most participants attended academic education for less than 12 years, with 11,888 people (52.70% of the total participants). Furthermore, 18,883 participants (83.70% of the total participants) were currently or ever married.

**TABLE 1:** Participants characteristics.

Variable	All	Men	Women	*P-*value
*N*	22,559 (100.00)	11,594 (51.39)	10,965 (48.61)	
Age (years), mean ± SD	38 ± 12	37 ± 12	38 ± 13	<0.001
Academic attainment, *n* (%)				<0.001
Less than 12 years	11,888 (52.70)	5,654 (48.77)	6,234 (56.85)	
More than equal to 12 years	10,671 (47.30)	5,940 (51.23)	4,731 (43.15)	
Matrimonial situation, *n* (%)				<0.001
Never married	3,676 (16.30)	2,266 (19.54)	1,410 (12.86)	
Currently or ever married	18,883 (83.70)	9,328 (80.46)	9,555 (87.14)	
Housing areas, *n* (%)				0.240
Rural	9,125 (40.45)	4,733 (40.82)	4,392 (40.05)	
Urban	13,434 (59.55)	6,861 (59.18)	6,573 (59.95)	
Smoking habit, *n* (%)				<0.001
Non-smoker	13,411 (59.45)	2,780 (23.98)	10,631 (96.95)	
Ex-smoker	1,006 (4.46)	936 (8.07)	70 (0.64)	
Smoker	8,142 (36.09)	7,878 (67.95)	264 (2.41)	
Food consumption score, mean ± SD	34.73 ± 14.78)	35.10 ± 14.76	34.34 ± 14.79	<0.001
Moderate PA volume (METs min/w), mean ± SD	1951.02 ± 2077.35)	2010.93 ± 2160.63	1894.32 ± 1993.84	<0.001
Vigorous PA volume (METs min/w), mean ± SD	4755.87 ± 4689.75)	5117.28 ± 4814.12	3483.47 ± 3970.80	<0.001
Waist circumference (cm), mean ± SD	84.64 ± 11.67)	83.16 ± 11.16	86.07 ± 11.98	< 0.001
Abdominal obesity^a,^ *n* (%)				<0.001
No	4,748 (51.10)	3,322 (72.74)	1,426 (30.19)	
Yes	4,543 (48.90)	1,245 (27.26)	3,298 (69.81)	
Body shape index (m^11/6^ kg^–2/3^), mean ± SD	0.0812 ± 0.0058	0.0806 ± 0.0050	0.0819 ± 0.0065	<0.001
Body mass index (kg/m^2^), mean ± SD	23.56 ± 4.43	22.58 ± 3.90	24.59 ± 4.71	<0.001
Body mass index classification^b^, *n* (%)				<0.001
<18.5	2,279 (10.10)	1,424 (12.28)	855 (7.80)	
18.5–25.0	12,792 (56.70)	7,359 (63.47)	5,433 (49.55)	
25.1–27.0	2,873 (12.74)	1,260 (10.87)	1,613 (14.71)	
>27.0	4,615 (20.46)	1,551 (13.38)	3,064 (27.94)	
Systolic blood pressures (mmHg), mean ± SD	128.82 ± 19.87	130.38 ± 17.51	127.17 ± 21.97	<0.001
Diastolic blood pressures (mmHg), mean ± SD	79.36 ± 12.05	79.46 ± 11.82	79.24 ± 12.30	0.171
Hypertension, *n* (%)				0.308
No	15,534 (68.86)	8,019 (69.17)	7,515 (68.54)	
Yes	7,025 (31.14)	3,575 (30.83)	3,450 (31.46)	
Cardiovascular diseases^c^, *n* (%)				<0.001
No	22,112 (98.02)	11,406 (98.38)	10,706 (97.64)	
Yes	447 (1.98)	188 (1.62)	259 (2.36)	

METs min/w, metabolic equivalent of tasks for minutes per week; PA, physical activity; SD, standard deviation. The categorical data were presented using n (%), and the continuous data were presented using mean ± SD.

^a^The definition of abdominal obesity for women and men was based on waist circumference with cutoff points > 80 cm or > 90 cm, respectively.

^b^The body mass index was calculated using the adult categorization of body mass index for the Indonesian population. ^c^Cardiovascular diseases are defined as the event of any stroke or cardiac heart disease that is diagnosed by the doctor. A significant p-value was set to <0.05.

Meanwhile, 13,411 participants (59.45% of the total participants) had never smoked. The physical activity volumes of moderate and vigorous exercise among women were lower than in men (*p* < 0.001). Moreover, the mean food consumption score among women (34.34 ± 14.79) was lower than that of men (35.10 ± 14.76), with a *p*-value less than 0.001. However, women had a higher mean body mass index, body shape index, and waist circumference than men. The number of women with abdominal obesity, classified as overweight or obese, and diagnosed with cardiovascular diseases was significantly higher than that of men (Table 1).

### Childhood socioeconomic status

Table 2 shows the distribution of childhood economic status by gender. Childhood socioeconomic status was measured when the participants were 12 years old. Childhood socioeconomic status was divided into three parts to simplify the table. First, the family situation consists of the participants’ family conditions, such as the parents’ marriage life, where and with whom the participants lived at that time and the job type of the breadwinner in the house. Second, parental behavior consisted of smoking habits, heavy alcoholic beverage consumption, and whether parents had mental problems. The last part was the participants’ housing situations, such as electricity and toilet ownership, drinking water source type, the number of books they owned, and the number of people and siblings who lived in the same dwelling.

**TABLE 2:** Distribution of childhood socioeconomic status.

Variable	All	Men	Women	*P-*value
Family situation				
Married biological parents				<0.001
No	2,133 (9.46)	1,006 (8.68)	1,127 (10.28)	
Yes	20,426 (90.54)	10,588 (91.32)	9,838 (89.72)	
Live with biological mother				0.041
No	1,604 (7.11)	785 (6.77)	819 (7.47)	
Yes	20,955 (92.89)	10,809 (93.23)	10,146 (92.53)	
Live with biological father				0.008
No	2,818 (12.49)	1,382 (11.92)	1,436 (13.10)	
Yes	19,741 (87.51)	10,212 (88.08)	9,529 (86.90)	
Live at the born place				0.284
No	18,171 (80.55)	9,307 (80.27)	8,864 (80.84)	
Yes	4,388 (19.45)	2,287 (19.73)	2,101 (19.16)	
Employment type of the breadwinner				0.100
Government/Private worker	6,056 (26.85)	3,169 (27.33)	2,887 (26.33)	
Self-employed	5,052 (22.39)	2,514 (21.68)	2,538 (23.15)	
Self-employed with temporary worker	8,012 (35.32)	4,173 (35.99)	3,839 (35.01)	
Self-employed with permanent worker	288 (1.28)	150 (1.29)	138 (1.26)	
Unpaid family worker	27 (0.12)	12 (0.10)	15 (0.14)	
Casual worker in agriculture	1,477 (6.55)	726 (6.26)	751 (6.85)	
Casual worker not in agriculture	1,387 (6.15)	722 (6.23)	665 (6.06)	
Transfer	144 (0.64)	70 (0.60)	74 (0.67)	
Pension	116 (0.51)	58 (0.50)	58 (0.53)	
Parental behaviors				
Smoke, *n* (%)				<0.001
No	7,096 (31.46)	3,417 (29.47)	3,679 (33.55)	
Yes	15,463 (68.54)	8,177 (70.53)	7,286 (66.45)	
Heavily alcohol beverages consumption, *n* (%)				0.011
No	22,459 (99.56)	11,530 (99.45)	10,929 (99.67)	
Yes	100 (0.44)	64 (0.55)	36 (0.33)	
Have mental problems, *n* (%)				0.043
No	22,548 (99.95)	11,585 (99.92)	10,963 (99.98)	
Yes	11 (0.05)	9 (0.08)	2 (0.02)	
Parental behaviors combination, *n* (%)				
Smoke + Heavily alcohol beverages consumption				<0.001
No	21,760 (96.46)	11,077 (95.54)	10,683 (97.43)	
Yes	799 (3.54)	517 (4.46)	282 (2.57)	
Smoke + Have mental problems				0.104
No	22,515 (99.80)	11,566 (99.76)	10,949 (99.85)	
Yes	44 (0.20)	28 (0.24)	16 (0.15)	
Smoke + Alcohol + Mental problems				0.198
No	22,546 (99.94)	11,585 (99.92)	10,961 (99.96)	
Yes	13 (0.06)	9 (0.08)	4 (0.04)	
Housing situation				
Having electricity, *n* (%)				0.158
No	10,000 (44.33)	5,192 (44.78)	4,808 (43.85)	
Yes	12,559 (55.67)	6,402 (55.22)	6,157 (56.15)	
Toilet ownership, n (%)				0.031
Yes, with septic tank	9,023 (40.00)	4,595 (39.63)	4,428 (40.38)	
Yes, without septic tank	3,443 (15.26)	1,784 (15.39)	1,659 (15.13)	
Shared toilet	894 (3.96)	428 (3.69)	466 (4.25)	
Public toilet	1,840 (8.16)	994 (8.57)	846 (7.72)	
Others	7,359 (32.62)	3,793 (32.72)	3,566 (32.52)	
Drinking-water source type, *n* (%)				0.030
Piped water	2,911 (12.90)	1,484 (12.80)	1,427 (13.01)	
Closed-well/Pump (Electric, Hand)	3,386 (15.01)	1,699 (14.65)	1,687 (15.39)	
Opened-well water	11,719 (51.95)	6,061 (52.28)	5,658 (51.60)	
Mineral water	615 (2.73)	286 (2.47)	329 (3.00)	
Others	3,928 (17.41)	2,064 (17.80)	1,864 (17.00)	
Number of book in the house, *n* (%)				0.068
None or very few (0–10 books)	18,344 (81.32)	9,382 (80.92)	8,962 (81.73)	
Enough to fill 1 shelf (11–25 books)	2,814 (12.47)	1,459 (??)	1,355 (12.36)	
Enough to fill 1 bookcase (26–100 books)	1,129 (5.00)	594 (5.12)	535 (4.88)	
Enough to fill 2 bookcases (101–200 books)	172 (0.76)	95 (0.82)	77 (0.70)	
Enough to fill 2 or more bookcases (more than 200 books)	100 (0.44)	64 (0.55)	36 (0.33)	
Number of people live in the same house, mean ± SD	6.80 ± 2.90	6.16 ± 2.32	6.33 ± 2.63	<0.001
Number of room in the house, mean ± SD	3.49 ± 1.75	3.61 ± 1.67	3.65 ± 1.64	0.070
Number of older brother, mean ± SD	0.82 ± 1.22	0.78 ± 1.06	0.75 ± 1.04	0.032
Number of older sister, mean ± SD	0.74 ± 0.99	0.72 ± 0.99	0.71 ± 1.02	0.455
Number of younger brother, mean ± SD	1.06 ± 1.23	0.75 ± 0.99	0.82 ± 1.02	<0.001
Number of younger sister, mean ± SD	1.02 ± 1.29	0.72 ± 0.95	0.78 ± 1.03	<0.001

SD, standard deviation. The categorical data were presented using number (%), and the continuous data were presented using mean ± SD. Significant p-value was set to < 0.05.

Furthermore, in the family situation, most of the participants’ parents were still married (*p* < 0.001). The number of participants who lived with their biological mothers was significantly higher among men (*n* = 10,809) than among women (*n* = 10,146), with a *p*-value of 0.041. The number of participants who lived with their biological fathers was also significantly higher among men than among women (*p* = 0.008). Regarding parental behaviors, the percentage of parents with smoking habits, mental health problems, or alcoholic beverage consumption was higher among male participants (*p* < 0.001) and (*p* = 0.011–0.043), respectively. The number of parents with alcoholic beverage consumption and mental health problems was low. The percentage of a combination of parental behavior (smoking habit and heavily alcoholic beverage consumption) was significantly higher among men (4.46%) than women (2.57%), with a *p*-value less than 0.001.

Furthermore, in the housing situation part, most participants had toilets with a septic tank in the house, while the drinking water source type was open-well water (*p* = 0.030–0.031). A toilet with a septic tank in a house is one of the standards of living in Indonesia. The number of people, books, older brothers, younger sisters, and younger brothers who live in the same dwelling as the participants was significantly different between male and female participants (*p* < 0.001–0.032).

### Association between childhood socioeconomic status and food security proxy

Table 3 shows the association between childhood socioeconomic status and food consumption scores. In this study, the food consumption score represents the food security proxy, named dietary diversity. In the family situation of childhood socioeconomic factors, a variable of biological parents who were still married was positively and significantly associated with the food consumption score in both crude and adjusted models (*p* < 0.001). The employment types of the breadwinners in the house were significantly associated with the food consumption score compared to the government/private worker type in both crude and adjusted models (*p* < 0.001–0.001), except for transfer and pension. The self-employed with permanent workers were positively associated (*p* = 0.001) with the outcome variable compared to the government/private workers, with exponentiated β-coefficients of 2.920 (95% CI: 1.188–4.652) in the crude model to 2.904 (95% CI: 1.172–4.636) in the adjusted model. Meanwhile, other employment types of breadwinners were negatively associated with the outcome variable (*p* < 0.001). Conversely, parents’ smoking habits in the parent’s behavior part were negative and significantly associated with the food consumption score (exponentiated β-coefficients of –0.540 (95% CI: –0.956 to –0.125, *p* = 0.011) in the crude model to –0.578 (95% CI: –0.993 to –0.162, *p* = 0.006) in the adjusted model). In contrast, the consumption of heavy alcoholic beverages and the combination of smoking and heavy alcoholic beverage consumption in parents’ behavior was positively and significantly associated with the food consumption score (*p* < 0.001–0.044).

**TABLE 3:** Regression model of the association between childhood socioeconomic status and outcome.

Variables	Crude				Adjusted			
		
	Coef.	95% CI		*p* value	Coef.	95% CI		*P*-value
Family situation								
Married biological parents (ref.: no)	1.402	0.743	2.061	<0.001	1.379	0.719	2.039	<0.001
Live with biological mother (ref.: no)	0.365	–0.385	1.116	0.340	0.348	–0.403	1.098	0.364
Live with biological father (ref.: no)	0.531	–0.052	1.115	0.074	0.515	–0.069	1.098	0.084
Live at the born place (ref.: yes)	–0.206	–0.694	0.281	0.406	–0.208	–0.696	0.280	0.404
Employment status of the breadwinner								
**Government/Private worker**	**Ref.**				**Ref.**			
Self-employed	–2.088	–2.635	–1.540	<0.001	–2.094	–2.642	–1.546	<0.001
Self-employed with temporary worker	–2.394	–2.883	–1.905	<0.001	–2.432	–2.923	–1.941	<0.001
Self-employed with permanent worker	2.920	1.188	4.652	0.001	2.904	1.172	4.636	0.001
Unpaid family worker	–9.650	–15.189	–4.110	0.001	–9.661	–15.200	–4.123	0.001
Casual worker in agriculture	–7.549	–8.382	–6.715	<0.001	–7.588	–8.424	–6.752	<0.001
Casual worker not in agriculture	–3.836	–4.691	–2.981	<0.001	–3.817	–4.672	–2.962	<0.001
Transfer	–1.559	–3.981	0.862	0.207	–1.469	–3.891	0.952	0.234
Pension	–0.287	–2.979	2.406	0.835	–0.299	–2.990	2.393	0.828
Parental behaviors								
Smoke (ref.: no)	–0.540	–0.956	–0.125	0.011	–0.578	–0.993	–0.162	0.006
Heavily alcohol beverages consumption (ref.: no)	3.066	0.164	5.969	0.038	2.983	0.080	5.886	0.044
Having mental problems (ref.: no)	–2.638	–11.373	6.098	0.554	–2.872	–11.606	5.862	0.519
Parental behaviors combination								
Smoke + Heavily alcohol beverages consumption (ref.: no)	1.964	0.921	3.007	<0.001	1.878	0.833	2.923	< 0.001
Smoke + Having mental problems (ref.: no)	–1.298	–5.669	3.073	0.561	–1.398	–5.768	2.972	0.531
Smoke + Alcohol + Mental problems (ref.: no)	3.506	–4.531	11.542	0.393	3.363	–4.671	11.397	0.412
Housing situation								
Having electricity (ref.: yes)	–2.791	–3.177	–2.404	<0.001	–4.007	–4.465	–3.550	< 0.001
Toilet ownership								
Yes, with septic tank	4.351	3.901	4.802	<0.001	4.955	4.479	5.430	< 0.001
Yes, without septic tank	0.137	–4.790	–3.640	0.651	0.318	–0.275	0.911	0.293
Shared toilet	1.257	–4.101	–2.089	0.015	1.576	0.558	2.593	0.002
Public toilet	1.103	–3.982	–2.514	0.004	1.263	0.515	2.011	0.001
**Others**	**Ref.**				**Ref.**			
Drinking-water source type								
Piped water	4.084	3.379	4.788	<0.001	4.421	3.707	5.134	< 0.001
Closed-well/Pump (Electric, Hand)	3.171	2.495	3.847	<0.001	3.586	2.897	4.276	< 0.001
Opened-well water	0.684	0.153	1.216	0.012	0.707	0.176	1.238	0.009
Mineral water	4.639	3.389	5.889	<0.001	5.447	4.170	6.724	< 0.001
**Other**	**Ref.**				**Ref.**			
Number of book in the house								
**None or very few (0–10 books)**	**Ref.**				**Ref.**			
Enough to fill 1 shelf (11–25 books)	3.624	3.043	4.205	<0.001	3.713	3.129	4.298	< 0.001
Enough to fill 1 bookcase (26–100 books)	7.267	6.387	8.147	<0.001	7.333	6.452	8.214	< 0.001
Enough to fill 2 bookcases (101–200 books)	7.676	5.478	9.874	< 0.001	7.701	5.504	9.898	< 0.001
Enough to fill 2 or more bookcases (more than 200 books)	7.912	5.035	10.789	< 0.001	7.846	4.970	10.722	< 0.001
Number of people live in the same house	0.224	0.146	0.301	< 0.001	0.231	0.152	0.309	< 0.001
Number of room in the house	0.939	0.823	1.055	< 0.001	0.968	0.850	1.085	< 0.001
Number of older brother	–0.012	–0.196	0.172	0.900	–0.018	–0.202	0.166	0.848
Number of older sister	0.129	–0.063	0.321	0.189	0.125	–0.068	0.317	0.204
Number of younger brother	0.350	0.158	0.541	< 0.001	0.368	0.173	0.563	< 0.001
Number of younger sister	0.198	0.003	0.392	0.046	0.209	0.011	0.407	0.039

The adjustment variables are age and gender. Significant p-value was set to < 0.05.

Regarding the housing situation of the childhood socioeconomic factors, owning an electricity facility in the house during childhood was significantly negatively associated with adulthood’s food consumption score in both regression models (*p* < 0.001). Toilet ownership (i.e., toilet ownership with a septic tank, public toilet, and shared toilet) was positively and significantly associated with food consumption scores (*p* < 0.001–0.015). The drinking water source type variable was positively and significantly associated with the food consumption score (*p* < 0.001–0.012). Furthermore, the number of books owned in the house was positively and significantly associated with the outcomes [exponentiated β-coefficients of 3.624–7.267 (95% CI: 3.043–8.147, *p* < 0.001)] in the crude model and the adjusted model (exponentiated β-coefficients of 3.713–7.333 (95% CI: 3.129–8.214, *p* < 0.001). Moreover, in the housing situation, the number of people who lived in the same dwelling was significantly positively associated with the food consumption score in both the crude and adjusted models, with exponentiated β-coefficients of 0.224 (95% CI: 0.146–0.301) to 0.231 (95% CI: 0.152–0.309), with *p* < 0.001. The food consumption score will increase by 0.224–0.231 units for every one-unit increase in the number of people who live in the same house as participants during childhood. The food consumption score increased by 0.939–0.968 units for every one-unit increase in the number of owned rooms in the participants’ houses when they were 12 years old (*p* < 0.001). Finally, the food consumption score increased by 0.198–0.209 units and by 0.350–0.368 units for every one-unit increase in the number of younger sisters and younger brothers who lived in the same house as the participants during childhood, respectively (*p* < 0.001–0.046).

## Discussion

The aim of this study was to assess the association between childhood socioeconomic status and food consumption scores. Most of the participants in this study were men aged <40 years, had low educational attainment, were married, or had ever had a marriage experience, and reported that they never had smoking habits. Furthermore, the food consumption score and physical activity volume were low among the female participants. Meanwhile, the mean body mass index, waist circumference, body shape index, and percentage of participants with abdominal obesity classified as overweight and diagnosed with CVD were high among women. Based on our study results, female participants are more likely to be food insecure, have less than 12 years of educational attainment, and have a disadvantaged health status (e.g., obesity and CVD). Previous researchers have suggested that vulnerable targets of food insecurity include women, people with low education levels, and people with low socioeconomic status, which leads to their poor health status (34–39). Low education levels lead to poor employment and low income, which leads to poverty and food insecurity among women, particularly those who live alone or are single parents (40–42). Among all the vulnerable targets, we still need to identify the determinant factors and see a prospective solution using multidisciplinary integrated approaches to solve food insecurity problems. Identifying determinant factors may start with socioeconomic status factors during childhood, which contributes to the experience of chronic food insecurity. This study assessed how socioeconomic status during childhood is associated with the food insecurity proxy or dietary diversity in adulthood.

Furthermore, childhood socioeconomic status was assessed using 16 questions and presented in three parts to simplify the reading results table and better understand the concept. Part one was the family part, which was about the situation of parental marriage-life during the participant’s life in childhood. Our study results showed that parents who were still married were positively and significantly associated with the food consumption score, which means that parental marital status change (i.e., becoming divorced, widowed, or separated) will decrease the food consumption score by 1.379 units. Children who live with married parents have a better chance of accessing various available foods. Parental socioeconomic status, including education, job, and financial factors, is related to parenting style, which may explain the association between parents’ marital status and the outcomes. The parents’ situation affects how they treat and feed their children. The healthy human body absorbs and utilizes adequate quality nutrients, which results in good health status. In addition, healthcare needs are related to individual health status, particularly for children, because they also need good nutrients to grow. Meanwhile, children with two parents are more likely to have met their healthcare needs than children with single mothers (43–45). Healthcare needs are important for all household members because they are related to food utilization (46), which is one of the pillars of food security.

Furthermore, parents’ socioeconomic status depends on their job type and income level. The job types in Indonesia commonly have stable income government/private workers. Another job type with a stable income is self-employed permanent workers, which shows a positive and significant association with the food consumption score. Our study participants’ parents with casual workers or other types of self-employed jobs were negatively and significantly associated with the food consumption score, with government/private workers as a reference in the parents’ job type regression analysis. The regression analysis showed that any changes in the parents’ job type, particularly in job types with an unstable income, will decrease the score of food consumption by 2.094–9.661 units. Financial resources and environmental food factors may affect the individual’s food security status (e.g., from the food access or food availability pillar) differently based on their geographical areas (47). Financial resources may be the key to food access. For example, although food is available, if the individual has difficulty buying or reaching the nearest food market, there will be a food insecurity problem. The difficulty in buying food may be affected by the type of breadwinner’s job and low or unstable income. A family with a stable income is more likely to have sustainable access to the available food in the market (48).

Moreover, self-employed workers or those self-employed with temporary workers may be more likely to receive unstable incomes because they do not guarantee their specific income. Although the size of the self-employed company may affect the income amount, when there is a fluctuation in the economic situation because of some issues in the political or social environment, these people may be more impacted than those with a stable income (48). However, the self-employed with permanent workers may provide a more stable income due to their ability to pay salaries regularly or monthly for their workers, which means they have enough benefits for themselves.

Meanwhile, casual workers (in or not in the agriculture sector) are associated with the climate, geography, and environmental situation. Some changes in climate or natural disasters affect agricultural results or harvest times (49, 50). The harvest amount and quality affect the worker’s income, which relates to the food access of food insecurity pillars if they sell the crops and will affect their food stock (related to food access and availability) if they consume it for themselves. An individual’s access to the variety of available foods in the market eventually affects their dietary diversity and food frequency.

Besides the parental situation, part two of the childhood socioeconomic factors was about the parents’ behavior. This study found that in the parental behavior part, smoking and alcoholic beverage consumption habits, in particular, were associated with the food security proxy. Parents’ smoking habits were negatively and significantly associated with their food consumption scores. In contrast, alcoholic beverage consumption habits and combination variables (smoking and alcohol consumption) were positively and significantly associated with the outcome variable. Smoking and alcoholic beverage consumption habits lead to chronic conditions such as hypertension and respiratory or cardiovascular diseases (51, 52). However, food insecurity is more prevalent among people with smoking habits (53–56). Some people may develop a smoking habit as a coping strategy in stressful situations, but buying tobacco products may also account for some proportion of the food expenditure, which leads to difficulty accessing a more diverse variety of available food for the family (57, 58). However, based on the majority religion of the Indonesian population, the consumption of alcoholic beverages is not a popular culture (59). The price of alcoholic beverages is high because of the additional tax that comes with it for both national and imported products. A family with financial ability can provide a greater proportion of non-food expenditure than food expenditure (60), which is less likely to happen among food insecure people, which may explain the positive association between alcoholic beverage consumption and the food security proxy in this study.

The third part of the childhood socioeconomic status factor was the participants’ housing situation during childhood. The house situation included any facility owned and the number of occupants in the same dwelling as the participants. One of the food insecurity indicators in the food security vulnerability atlas is the percentage of households with access to electricity (61). A person without access to electricity in their house was negatively associated with the food security proxy in our study results. Furthermore, electricity is essential for people to maintain their food in a refrigerator or room at a controllable temperature. Another benefit of electricity is street lighting, in-house lighting, or other electronic devices to support food supply, preparation, production, and distribution systems. A family with electricity needs to spare some of its income to pay for the electricity bill. Thus, families with better or stable incomes may pay this bill without participating in food expenditure. Based on the electricity benefit of providing the power for refrigerators to prolong the shelf life of various food types, which leads to a more diverse diet, people with food security are more likely to receive benefits from it than food insecure ones.

Furthermore, one of the food security pillars is food utilization, which is about how a healthy person’s body utilizes nutrients from food, which results in good health. To maintain an optimal health status related to food security, we must prevent food utilization problems, such as use of safe and good quality water for drinking and preparing food, or hygiene sanitation for all household members. Poor water quality and hygienic sanitation can be sources of infectious diseases that lead to food utilization problems. The infection disrupts food utilization in the body and lowers the diversity of the consumed diet due to the lack of appetite, which eventually results in malnutrition in children, such as stunting (62, 63), which has long-term effects on children’s lives. Water quality (i.e., physical, chemical, and microbiological) and water safety, which may have immediate health consequences, play an important role in infectious disease prevention (64). The quality of the water in the house for food preparation leads to good food utilization with a more diverse consumed diet, which may explain the positive association between safe drinking water source types and the outcome variable in our study.

Moreover, in hygiene sanitation management, toilet ownership must meet the standard to prevent fecal contamination through soil water, which is dangerous to the drinking water source of the house. The standards for toilet ownership must have a septic tank. Meanwhile, this study results showed that house situations with toilets, septic tanks, and shared or public toilets were positively and significantly associated with the food consumption score. However, public or shared toilets may help them access the toilet facility together because not all people can afford to have proper toilets in their houses or those who live under the poverty level. Public or shared toilets usually meet the standard and have hygiene sanitation facilities that prevent infectious diseases, which may explain the positive association between toilet-type ownership and the outcome variable.

A positive association was also found in the housing situation between the number of books owned and the outcomes of this study. The number of books owned in the house may relate to better education and literacy level of the household members or a higher non-food expenditure that is more likely to happen among food-secure people with no problem fulfilling a diverse diet. In contrast, although the study result showed a positive association between the number of owned rooms in the house, the number of people, younger sister, younger brother, and food consumption scores, the exponentiated beta value was less than one, which means that the association is weaker than the beta value that is larger than one. The increased number of rooms in the house may be related to the presence of additional family members. A family can increase the number of rooms in their house if they have sufficient money, which means that their food expenditure is not affected by house renovation fees. A family with five or more people in a house is considered large. One of the determining factors of urban household food insecurity is the large size of the family (65). The number of people or younger siblings living in the same dwelling represents the size of the family. Family size grows when younger siblings are born, and parents already prepare savings for the new family member, which means they have more than enough money for food and non-food expenditure. A stable financial situation leads to better access to a more diverse diet that can be purchased in the market.

Our study has some limitations. The childhood socioeconomic status questionnaire was prone to recall bias, which may potentially lead to underestimation of the association between variables. However, the questionnaire has been widely used in previous research (33, 66–68). Another limitation was that the food consumption score did not represent all food insecurity proxies. However, food consumption score analyses are widely used for food security assessment as a composite score of dietary diversity and food frequency in developing countries (15, 16, 29, 69). Further research should consider additional information on food recall to provide complete information on dietary diversity related to nutrient adequacy. In addition, more variables that become possible proof of childhood socioeconomic status, such as the ownership of tertiary products (i.e., a refrigerator and a vehicle that is not used for work), should be included.

## Conclusion

In respective order among the childhood socioeconomic status factors, the number of owned books, the use of safe drinking-water sources and standard toilets, parents with alcohol consumption habits or a combination of smoking habits, self-employed permanent workers, still married biological parents, the number of rooms, people, and younger siblings were positively and significantly associated with the food security proxy. Contrarily, in respective order among the childhood socioeconomic status factors, self-employment without permanent workers and casual work types, houses with electricity facilities, and parents with smoking habits were negatively and significantly associated with the outcomes. Therefore, children with early socioeconomic disadvantages may experience chronic food insecurity, which affects their adult food security status and leads to poor health status. Thus, integrated work with all sectors may consider food insecure adults with the possibility of chronic food insecurity as the priority of vulnerable targets of food and nutrition security improvement programs.

## Data availability statement

Publicly available datasets were analyzed in this study. This data can be found here: http://www.rand.org/labor/FLS/IFLS/download.html#updates.

## Ethics statement

The studies involving human participants were reviewed and approved by RAND’s Human Subjects Protection Committee, RAND Corporation. The patients/participants provided their written informed consent to participate in this study.

## Author contributions

EI: conceptualization, methodology, and writing—original draft preparation. EI, Y-CC, and S-HY: writing—review and editing. S-HY: supervision. All authors read and agreed to the published version of the manuscript.
