# Supplementary material for: Childhood socioeconomic status and adulthood dietary diversity among Indonesian adults
Source: Front Nutr. 2022 Sep 23;9:948208. doi: 10.3389/fnut.2022.948208 (PMC9537565; doi:10.3389/fnut.2022.948208)
Supplement: Supplementary file 1 [file Data_Sheet_1.docx]

Supplementary Material

# Supplementary Table

Table S1. Childhood economic status questionnaire of IFLS 2014

| **No.** | **Question** | **Rate** |
| --- | --- | --- |
| A | When you were 12, were your biological parents still married? | Yes/No/NA |
| B | When you were 12, did you live with your biological mother? | Yes/No/NA |
| C | When you were 12, did you live with your biological father? | Yes/No/NA |
| 1 | When you were 12, how many rooms did your household occupy in the accommodation, including bedrooms but excluding kitchen, bathrooms, and hallways?  *Interviewer: Do not count box room, cellar, attic, etc.* | Numbers |
| 2 | Including yourself, how many people lived in your household at this accommodation when you were 12? | Numbers |
| 3 | How many older brothers lived in your household at this accommodation when you were 12? | Numbers |
| 4 | How many older sisters lived in your household at this accommodation when you were 12? | Numbers |
| 5 | How many younger brothers lived in your household at this accommodation when you were 12? | Numbers |
| 6 | How many younger sisters lived in your household at this accommodation when you were 12? | Numbers |
| 7 | When you were 12, did any of your parents:   1. Smoke 2. Drink Heavily 3. Have Mental Problems 4. Smoke + Drink Heavily 5. Smoke + Have Mental Problems 6. Smoke + Drink Heavily + Have Mental Problems | Yes/No  Yes/No  Yes/No  Yes/No  Yes/No  Yes/No |
| 8 | When you were 12, did you live in the same place when you were born? | Yes/No/NA |
| 9 | When you were 12, did your households utilize electricity? | Yes/No |
| 10 | When you were 12, what is the main water source for drinking in your household? | - Piped water - Closed-Well/pump (electric, hand) - Opened-Well water - Mineral Water - Other |
| 11 | When you were 12, where do the majority of householders go to the toilet? | - Own toilet with septic tank - Own toilet without septic tank - Shared toilet - Public toilet - Other |
| 12 | Approximately how many books were there in the place you lived in when you were 12?  *Interviewer: Do not count magazines, newspapers, or your school books.* | - None or very few (0-10 books) - Enough to fill 1 shelf (11-25 books) - Enough to fill 1 bookcase (26-100 books) - Enough to fill 2 bookcases (101-200 books) - Enough to fill 2 or more bookcases (more than 200 books) |
| 13 | What best describes the employment status of the household's main breadwinner when you were 12?  *Interviewer: The main breadwinner is the person providing the majority of income for the household.* | - Unpaid family worker - Self-employed - Self-employed with unpaid family worker/temporary worker - Self-employed with permanent worker - Government worker - Private worker - Casual worker in agriculture - Casual worker not in agriculture |

Note: NA, not applicable; IFLS, Indonesia Family Life Survey.

# Supplementary Figure

Figure S1. Flowchart of selection participants.

IFLS5 BUS_US data (all ages: 0 to ≥ 80 years)

Person with disability (289)

Missing data on weight, height (327)

Women with who were pregnant and
breastfeeding (2499)

Age younger than 20 years, or older than 65 years (19653); Person with cancer (161)

Missing data on food frequency questionnaire, childhood socioeconomic status (2651)

48,139

47,523

45,024

25,210

22,559
